# A gene-wide investigation on polymorphisms in the taste receptor 2R14 (*TAS2R14*) and susceptibility to colorectal cancer

**DOI:** 10.1186/1471-2350-11-88

**Published:** 2010-06-09

**Authors:** Daniele Campa, Pavel Vodicka, Barbara Pardini, Alessio Naccarati, Maura Carrai, Ludmila Vodickova, Jan Novotny, Kari Hemminki, Asta Försti, Roberto Barale, Federico Canzian

**Affiliations:** 1 German Cancer Research Center (DKFZ), Heidelberg, Germany; 2 Department of Biology, University of Pisa, Pisa, Italy; 3 Institute of Experimental Medicine, Academy of Sciences of the Czech Republic, Prague, Czech Republic; 4 Department of Oncology, First Faculty of Medicine, Charles University, Prague, Czech Republic

## Abstract

**Background:**

Molecular sensing in the gastro-intestinal (GI) tract is responsible for the detection of ingested harmful drugs and toxins, thereby genetic polymorphisms affecting the capability of initiating these responses may be critical for the subsequent efficiency of the gut in eliminating possible threats to the organism. Although these fundamental control systems have been known for long time, the initial molecular recognition events that sense the chemical composition of the luminal contents of the GI tract have remained elusive. TAS2R14 is one of the better characterized members of the taste receptor family and has several polymorphic variants. Several substances that have been shown to activate TAS2R14 are powerful toxic and carcinogenic agents.

**Methods:**

Using a tagging approach we investigated all the common genetic variation of the gene region in relation to colon cancer risk with a case-control study design. This is, at the best of our knowledge also the first report on the allele frequencies of the gene in the Caucasian population.

**Results:**

We found no evidence of statistically significant associations between polymorphisms in the *TAS2R14 *gene and colon cancer risk.

**Conclusion:**

In conclusion we can confidently exclude a major role for common polymorphisms of the *TAS2R14 *gene in colorectal cancer risk in this population, although in this report we had insufficient statistical power to completely exclude the possibility that rare variants of the TAS2R14 might be involved in colorectal cancer risk.

## Background

The gustatory system has been selected during evolution to detect non-volatile nutritive and beneficial compounds as well as potentially harmful substances. In particular, bitter taste has evolved as a central warning signal against the ingestion of potentially toxic substances, including plant alkaloids and other environmental toxins. Recently, the most studied family of bitter taste receptors (referred as TAS2Rs) [1] has been characterized not just in the tongue but also in the gut[2]. The gastrointestinal (GI) mucosa comes in direct contact with a vast array of potentially harmful substances in the lumen and acts as a sensory organ by detecting luminal content and sending messages to the nervous system to initiate the appropriate response of neutralization and expulsion of drugs, toxins, and microorganisms. While it is true that certain ligands that activate TAS2Rs are biotransformed to eliminate their toxicity, the biotransformation of other ligands can increase toxicity. The first, and perhaps strongest, line of defense against toxins may be at the level of taste by avoiding ingestion.

As a second line of defence molecular sensing in the GI tract could be responsible for the detection of ingested harmful drugs and toxins, thereby initiating responses critical for survival. Furthermore, molecular sensing of luminal contents also initiates hormonal and/or neural pathways leading to the regulation of caloric intake, pancreatic insulin secretion, and metabolism [3].

Although these fundamental control systems have been known for a considerable amount of time, the initial molecular recognition events that sense the chemical composition of the luminal contents of the GI tract have remained elusive. Indeed, the receptors and signaling pathways that transduce the effects of many luminal molecules have not been identified, and the cellular and neural pathways that mediate biological responses to luminal stimuli in general, and bitter stimuli in particular, remain poorly characterized.

In humans, bitter taste is mediated by approximately 30 G protein-coupled receptors belonging to the TAS2R gene family [4-6]. Some of TAS2Rs receptors are narrowly tuned for their corresponding ligands. However recent reports have shown that several, including TAS2R14, have extremely broad spectra for activation towards a large variety of putative bitter tastants that are structurally very divergent[7,8]. This feature of TAS2R14 might explain how the limited number of bitter taste receptors enables mammals to recognise thousands of different bitter tasting molecules.

Several bitter substances that have been shown to activate TAS2R14 are powerful toxic agents like picrotoxin, which is an antagonist of GABAA-receptors leading to neurotoxic effects including convulsant action in human and rodents [7] and aristolochic acid, which is a strong carcinogen that nonetheless is used as an herbal remedy for weight loss. These findings suggest that this receptor indeed is a functional warning sensor of the concentrations of potentially harmful compounds within food containing those substances.

Genetic variants affecting the capability of initiating these responses may be critical for the subsequent efficiency of the gut in eliminating possible threats to the organism[3]. *TAS2R14 *is one of the better characterized members of the T2R family and has several polymorphic variants. Although functional studies did not elucidate the biological relevance of the single nucleotide polymorphisms (SNPs) in this gene, several studies have been conducted in the other members of the T2R family with promising results [9-11].

We conducted a study to investigate the hypothesis that genetic variants of TAS2R14 could influence its ability to bind bitter toxic compound, initiate the elimination of xenobiotic and thus increase the risk of colorectal cancer. Using a tagging approach we covered all the genetic variation of the gene, and tested tagging SNPs in 680 cases of colon cancer and 590 controls.

## Methods

### Study population

For the present case-control study, six oncological and five large gastroenterological departments in various regions of the Czech Republic contributed by providing blood samples and anamnestic data. The study is based on incident cases, whose recruitment started in September 2004 and finished in February 2006.

Cases consist of patients with positive colonoscopic results for malignancy, histologically confirmed as colon or rectal carcinomas. In the group of cases, genetic testing for hereditary HNPCC was recommended to four patients, who belonged to families complying with the Amsterdam criteria II. These patients were excluded from our study.

Controls were sampled at the same time as cases, and chosen among subjects undergoing colonoscopy due to macroscopic bleeding, positive fecal occult blood test, or abdominal pain of unknown origin. The most common findings for these subjects were hemorrhoids or idiopatic bowel diseases (IBD). Only subjects whose colonoscopic results were negative for malignancy, colorectal adenomas or IBD were chosen as controls.

Study subjects provided information on their lifestyle habits (smoking, drinking, diet etc.), tentative occupational exposure to xenobiotics, and family/personal history of cancer, with the use of structured questionnaires [12,13].

We included in this study 680 cases and 590 controls. The relevant characteristic of the studied population are shown in table 1. The genetic analyses did not interfere with diagnostic or therapeutic procedures for the subjects. All participants signed an informed written consent and the design of the study was approved by the Ethical Committee of the Institute of Experimental Medicine, Prague, Czech Republic.

**Table 1:** Characteristics of colorectal cancer patients and control subjects

		Cases	Controls
		(*n *= 680)	(*n *= 590)
Males/Females (%)*		57.2/48.2	53.6/46.4

**Age**	**Average**	61.1 yrs	56.0 yrs

	**Lower Quartile**	55 yrs	47 yrs

	**Upper quartile**	69 yrs	66 yrs

**Rectal cancer (%)**		38.4	
**Colon cancer (%)**		61.7	

Smoking*	**Non smokers (%)**	53.6	52.8
	**Ex-smokers (%)**	32.3	26.4
	**Smokers (%)**	14.1	20.9

Alcohol consumption*	**no (%)**	47.3	40.2
	**yes (%)**	52.7	59.8

Mean g of alcohol/day*		23.1	22.9

Living Place*	**City (%)**	57.2	56.2
	**Suburbs (%)**	15.5	21.2
	**Country (%)**	27.3	22.7

Education*	**Basic (%)**	33.5	25.4
	**High School (%)**	51.6	54.8
	**University (%)**	14.9	19.8

Mean BMI (Kg/m^2^) ± SD*		26.7 ± 4.3	26.7 ± 4.5

Distribution of the populations according to BMI:*	**< 18.5 Kg/m^2 ^(%)**	1.56	0.73
	**18.5-24.9 Kg/m^2 ^(%)**	37.0	37.9
	**25-29.9 Kg/m^2 ^(%)**	42.5	41.5
	**30-34.9 Kg/m^2 ^(%)**	15.1	15.3
	**≥ 35 Kg/m^2 ^(%)**	3.80	4.61

* No statistically significant differences were found for these covariates, between cases and controls

### Selection of tagging SNPs

**Figure 1:**
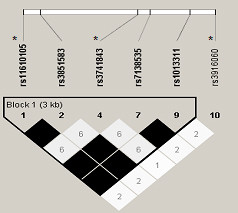
***TAS2R14 *locus and its tagging SNPs**. From top to bottom: SNPs genotyped in HapMap, graphical representation of LD and block structure (darker color represents higher LD, numbers in the colored squares are percentage of LD, expressed as r^2^; absence of number means r^2 ^= 100%). Asterisks denote tagging SNPs selected for genotyping.

We aimed at surveying the entire set of common genetic variants in *TAS2R14*. To this end, we followed a tagging approach. We used the algorithm described by Carlson and coworkers [14] that was developed to select the maximally informative set of tagSNPs in a candidate-gene association study. All polymorphisms in the region of *TAS2R14 *(including 5 kb upstream of the first exon and 5 kb downstream of the last exon) with minor allele frequency (MAF) ≥ 5% in Caucasians from the International HapMap Project (version 21a; http://www.hapmap.org) were included. Tagging SNPs were selected with the use of the Tagger program within Haploview http://www.broad.mit.edu/mpg/haploview/; http://www.broad.mit.edu/mpg/tagger/[15,16], using pairwise tagging with a minimum r^2 ^of 0.8. This resulted in a selection of 3 tagging SNPs, with a mean r^2 ^of the selected SNPs with the SNPs they tag of 1, meaning that our selection captures completely the known common variability in this gene. Considering that the genomic region of *TAS2R14 *is characterized by high levels of linkage disquilibrium (LD), we postulate that such SNPs are also likely to tag any hitherto unidentified common SNPs in the gene. Figure 1 shows *TAS2R14 *and its selected tagging SNPs.

### DNA extraction and genotyping

DNA was extracted from blood samples with standard proteinase K digestion followed by phenol/chloroform extraction and ethanol precipitation. The order of DNAs of cases and controls was randomized on PCR plates in order to ensure that an equal number of cases and controls could be analyzed simultaneously. All genotyping was carried out using the Taqman assay. The MGB Taqman probes and primers were purchased from Applied Biosystems (Foster City, CA) as pre-designed assays. The reaction mix included 10 ng genomic DNA, 10 pmol each primer, 2 pmol each probe and 2.5 ml of 2× master mix (Applied Biosystems) in a final volume of 5 µl. The thermocycling included 40 cycles with 30 s at 95°C followed by 60 s at 60°C. PCR plates were read on an ABI PRISM 7900HT instrument (Applied Biosystems).

All samples that did not give a reliable result in the first round of genotyping were resubmitted to up to two additional rounds of genotyping. Data points that were still not filled after this procedure were left blank. Repeated quality control genotypes (8% of the total) showed an average concordance of 99.9%.

### Statistical Analysis

The frequency distribution of genotypes was examined for the cases and the controls. Hardy-Weinberg equilibrium was tested in the cases and in the controls separately by chi square test. We used logistic regression for multivariate analyses to assess the main effects of the genetic polymorphism on CRC risk using a codominant inheritance model. The most common allele in the controls was assigned as the reference category. All analyses were adjusted for age and sex.

Additionally, we performed a logistic regression stratifying for the cancer site (colon *versus *rectum), smoking habits (smokers *versus *non smokers and heavy smokers *versus *light smokers (were considered heavy smokers those individuals that smoked more than 20 cigarettes a day), alcohol consumption and BMI (both considered as a continuous variable).

All analyses were performed with STATA software (StataCorp, College Station, TX).

## Results

Cases and controls were similar in terms of gender distribution. We analyzed the frequencies and distributions of the genotypes in cases and in controls. The genotype distributions at all loci were in Hardy-Weinberg equilibrium in controls, with non-significant chi square values (p < 0.05, data not shown).

We used the Tagger software to check the extent of LD between the SNPs we selected. As expected, we found very low LD between the selected tagging SNPs (Figure 1). The frequencies and distribution of the genotypes and the odds ratios for the association of each polymorphism with colorectal cancer risk are described in Table 2.

**Table 2:** Associations of *TAS2R14 *tagging polymorphisms with colorectal cancer risk

	Position on Chromosome 12	Cases^a^	Controls^a^	OR (95%)^b^	P value	P trend
**rs3916060**	10,984,340					
T/T		603	537	1		1.00
T/C		52	50	0.93 (0.62-1.39)	0.71	
C/C		2	0	N.A.	N.A.	
T/C+C/C		54	50	0.96 (0.64-1.44)	0.85	
						
**rs3741843**	10,982,699					
A/A		509	450	1		0.32
A/G		143	109	1.11 (0.43-2.82)	0.83	
G/G		10	8	1.16 (0.88-1.53)	0.30	
A/G+GG		153	117	1.16(0.88-1.52)	0.30	
						
**rs11610105**	10,980,242					
G/G		304	283	1		0.75
A/G		275	239	0.84(0.56-1.25)	0.39	
A/A		55	61	1.07(0.84-1.36)	0.57	
A/G+GG		330	300	1.02(0.82-1.28)	0.84	

^a ^Numbers may not add up to 100% of subjects due to genotyping failure. All samples that did not give a reliable result in the first round of genotyping were resubmitted to up to two additional rounds of genotyping. Data points that were still not filled after this procedure were left blank.

^b ^OR: odds ratio; CI: confidence interval. Adjusted for age and gender.

We found no statistically significant association between *TAS2R14 *SNPs and colorectal cancer risk.

Since several polymorphism in T2R genes have been found to be associated to behavioural traits like smoking habit and alcohol consumption or anthropometric measures like body mass index (BMI), and these variables are considered to be risk factors for colorectal cancer, we also performed analysis on possible association between the tagging SNPs and BMI, alcohol consumption, smoking behaviour We also stratified the analysis by cancer location (colon *vs *rectum) and measured the above mentioned variables both separately for cases and controls and pooling them together.

We found that carriers of the G allele of the SNP rs11610105 were more likely to be light smokers rather than being heavy smokers (number of cigarettes/day> = 20), with an odds ratio of 0.31 (95% confidence interval 0.16-0.64, P = 0.001). This association was observed only in the heterozygous individuals and just in controls.

We did not find any other association between the *TAS2R14 *gene polymorphisms and the measured endpoints.

## Discussion

Bitter taste has evolved as a central warning signal against the ingestion of potentially toxic substances, including plant alkaloids and other environmental toxins. Recently, TAS2R family members have been characterized in the gut [2]. The GI mucosa comes in direct contact with a vast array of potentially harmful substances in the lumen and acts as a sensory organ and is, therefore, responsible for the detection of ingested harmful drugs and toxins. Thereby SNPs affecting the capability of initiating these responses may be critical for the subsequent efficiency of the gut in eliminating possible threats to the organism[3]. Furthermore, molecular sensing of luminal contents also initiates hormonal and/or neural pathways leading to the regulation of caloric intake, pancreatic insulin secretion, and metabolism [3].

Several TAS2Rs receptors are narrowly tuned for their corresponding ligands. However recent reports have shown that several, including TAS2R14, have extremely broad spectra for activation. towards a large variety of putative bitter tastants that are structurally very divergent.

TAS2R14 has been shown to be activated by various substances, several of which are powerful toxic agents like picrotoxin and aristolochic acid which is a strong carcinogen, that nonetheless is used as an herbal remedy for weight loss.

In this case-control study, we explored the role of three tagging polymorphisms in the *TAS2R14 *gene. Since bitter taste sensitivity has been reported to be associated with smoking and alcohol consumption [17-19], we explored at the same time the association of *TAS2R14 *common variants with anthropometric measures, behavioural traits like smoking and alcohol consumption and colorectal cancer susceptibility.

A gene-wide tagging approach is currently accepted as the model for genetic association studies [20]. We selected tagging SNPs in order to cover all the genetic variance of *TAS2R14 *with a minimum threshold of r^2^>0.8 and MAF >0.05. However, the real r^2 ^with which our tagging SNPs tagged the other polymorphic variants in the gene was 1. Therefore we covered all the common (MAF>0.05) known genetic variability of this gene achieving thus a complete survey of the possible association between the polymorhisms and the endpoints taken into account.

We found no evidence of statistically significant associations between SNPs in the TAS2R14 gene and colorectal cancer risk.

We found that carriers of the G allele of the SNP rs11610105 were more likely to be light smokers than being heavy smokers. This finding was observed only in the heterozygous individuals and only in controls, and could thus be appealing because smoking habits and in particular heavy smoking is a well known risk factor for many cancer types. However, the finding was observed in a population subgroup of a relatively small size (n = 167), therefore this could be a chance finding and it has to be taken with caution.

## Conclusion

In conclusion we can confidently exclude a major role for common polymorphisms of the *TAS2R14 *gene in colorectal cancer risk in this population, although in this report we had insufficient statistical power to completely exclude the possibility that rare variants of the TAS2R14 might be involved in colorectal cancer risk.

## Competing interests

The authors declare that they have no competing interests.

## Authors' contributions

DC carried out the genotyping the statistical analysis and drafted the manuscript RB conceived the study and FC supervised the genotyping and the SNPs selection, PV, BP, AN, MC, LV, JN enrolled the subjects of the study and helped in the manuscript writing, KH, AF helped in writing the manuscript. All authors read and approved the manuscript

## Pre-publication history

The pre-publication history for this paper can be accessed here:

http://www.biomedcentral.com/1471-2350/11/88/prepub
